# Characteristics of Snakebite-Related Infection in French Guiana

**DOI:** 10.3390/toxins14020089

**Published:** 2022-01-24

**Authors:** Stéphanie Houcke, Dabor Resiere, Guy Roger Lontsingoula, Fabrice Cook, Pierre Lafouasse, Jean Marc Pujo, Magalie Demar, Severine Matheus, Didier Hommel, Hatem Kallel

**Affiliations:** 1 Intensive Care Unit, Cayenne General Hospital, 97300 Cayenne, French Guiana, France; stephanie.houcke@ch-cayenne.fr (S.H.); dabor.resiere@chu-martinique.fr (D.R.); guy.lontsingoulla@ch-cayenne.fr (G.R.L.); fabrice.cook@ch-cayenne.fr (F.C.); pierre.lafouasse@ch-cayenne.fr (P.L.); severine.matheus@ch-cayenne.fr (S.M.); didier.hommel@ch-cayenne.fr (D.H.); 2 Intensive Care Unit, Martinique University Hospital, 97261 Martinique, France; 3 Emergency Department, Cayenne General Hospital, 97300 Cayenne, French Guiana, France; jean.pujo@ch-cayenne.fr; 4 Laboratory Department, Cayenne General Hospital, 97300 Cayenne, French Guiana, France; magalie.demar@ch-cayenne.fr

**Keywords:** snakebite envenoming, clinical manifestations, French Guiana, *Bothrops atrox*, infection

## Abstract

Wound infection is frequently reported following snakebite (SB). This study is retrospective. It was conducted in the emergency department and the Intensive Care Unit (ICU) of Cayenne General Hospital between 1 January 2016 and 31 July 2021. We included 172 consecutive patients hospitalized for SB envenoming. All patients were monitored for wound infection. Sixty-three patients received antibiotics at admission (36.6%). The main antibiotic used was amoxicillin–clavulanate (92.1%). Wound infection was recorded in 55 cases (32%). It was 19% in grade 1, 35% in grade 2, and 53% in grade 3. It included abscess (69.1%), necrotizing fasciitis (16.4%), and cellulitis (21.8%). The time from SB to wound infection was 6 days (IQR: 3–8). The main isolated microorganisms were *A. hydrophila* and *M. morganii* (37.5% and 18.8% of isolated organisms). Surgery was required in 48 patients (28.1%), and a necrosectomy was performed on 16 of them (33.3%). The independent factors associated with snakebite-associated infection were necrosis (*p* < 0.001, OR 13.15, 95% CI: 4.04–42.84), thrombocytopenia (*p* = 0.002, OR: 3.37, 95% CI: 1.59–7.16), and rhabdomyolysis (*p* = 0.046, OR: 2.29, 95% CI: 1.02–5.19). In conclusion, wound infection following SB is frequent, mainly in grade 2 and 3 envenomed patients, especially those with necrosis, thrombocytopenia, and rhabdomyolysis. The main involved bacteria are *A. hydrophila* and *M. morganii*.

## 1. Introduction

Snake envenoming is a public health problem in French Guiana [1,2]. It can be responsible for systemic and local manifestations. Local manifestations start within minutes after the snakebite (SB). They are associated with pain and edema and are followed, in severe cases, by local necrosis and blistering, which can turn into infection [3]. In French Guiana, the incidence of wound infection (WI) secondary to SB and the responsibility of microorganisms remains poorly investigated. Thus, local recommendations are only based on data from other regions and concerning other snakes [4,5,6,7].

Wound infection following snakebite occurs in 9–77% of the bitten patients [5,6,8,9,10,11]. The main responsible microorganisms are *Aeromonas hydrophila*, *Morganella morganii*, *Klebsiella pneumoniae*, *Bacillus* spp., and *Enterococcus* spp. [6,12]. Bacteria are inoculated into the wound from the snake’s oral cavity, along with the venom. Furthermore, local skin and muscle damages constitute a favorable field for bacterial growth. For this, some authors questioned the relevance of empiric antibiotic therapy in snake-bitten patients [7].

We conducted this retrospective study to assess the prevalence of wound infection after snake envenoming in French Guiana, to identify the involved bacteria, and to monitor the use of antibiotics in this context.

## 2. Results

From 1 January 2016 to 31 July 2021, 172 patients were admitted with a diagnosis of snakebite envenoming (an average of 31 cases per year). The median age of patients was 41 years (IQR: 28–52), and 69% were male. The median time from snakebite to hospital admission was 09:00 (IQR: 02:00–21:00), and the median time between snakebite and antivenom administration was 9:00 (IQR: 5:22–20:40). Snake identification was made in 45.3% of cases. It was *Bothrops atrox* in 66 patient (84.6% of identified snakes). During the follow-up, 55 cases (32%) developed wound infection.

### 2.1. Clinical and Biological Manifestations at Admission

At admission, the main clinical symptoms were edema (95.9%), pain (96.5%), systemic bleeding (14%), blister (12.8%), and local hemorrhage (12.8%). The elapsed time from snakebite to the development of systemic bleeding was 5 h (IQR: 0–24). The comparison of the two groups with and without wound infection showed a higher prevalence of necrosis (30.9% vs. 3.4%) and a higher cardiac rhythm (90 vs. 79 beats/min) in the wound infection group (*p* < 0.001 and 0.002, respectively). Table 1 summarizes the epidemiological and clinical parameters of our patients.

Biologic parameters at admission showed defibrinogenation in 151 cases (87.8%), thrombocytopenia in 49 cases (28.5%), hemolysis in 38 cases (22.1%), and rhabdomyolysis in 54 cases (31.4%). Thrombocytopenia and rhabdomyolysis were more frequent in the wound infection group (Table 1).

### 2.2. Initial Management

Initial management was based on analgesics (100%), fluid infusion (73.3%), blood components transfusion (9.9%), dialysis (5.2%), mechanical ventilation (1.7%), and norepinephrine infusion (1.2%). Sixty-three patients (36.6%) received antibiotics at admission. Amoxicillin–clavulanate was the main antibiotic used (92.1% of patients who received antibiotics at admission). The duration of initial amoxicillin–clavulanate administration was 1 day (IQR: 1–1 days). Antivenom was prescribed for 115 patients (66.9%), and 20 of them (17.2%) developed early adverse reactions. They were mild in 13 cases (11.3%) and severe in 7 cases (6.1%). 

### 2.3. Wound Infection

**Figure 1:**
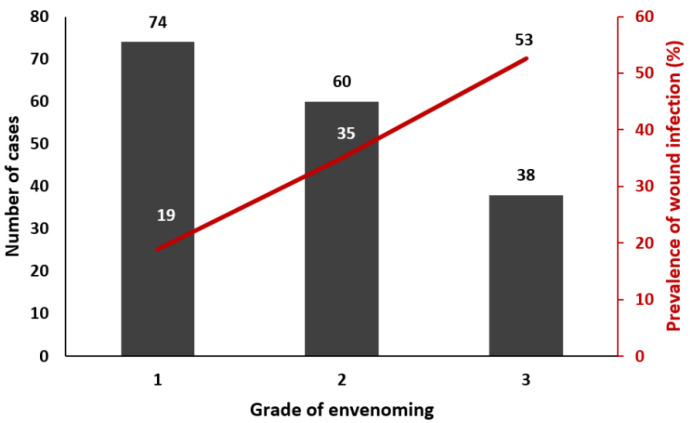
Prevalence of wound infection following snakebite according to the grade of envenoming.

**Figure 2:**
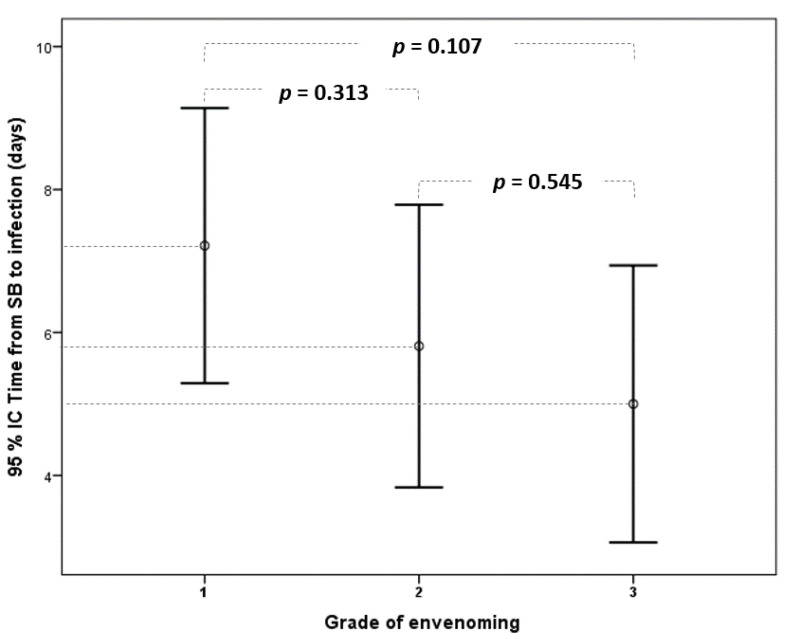
Comparison of elapsed time from snakebite to the diagnosis of wound infection according to the grade of envenoming. (95% CI: 95% confidence interval).

Wound infection was observed in 19% of cases in grade 1 (14/74), 35% in grade 2 (21/60), and 53% in grade 3 patients (20/38) (Figure 1). It included abscess (69.1%), necrotizing fasciitis (16.4%), and cellulitis (21.8%) (Table 2). The time from snakebite to infection was, on average, 6 days (IQR: 3–8). There was no statistical difference according to the grade of envenoming (Figure 2).

### 2.4. Microbiological Analyses

Microbiological analysis was positive in 32 patients (58.2%). It was positive in 9.5%, 20%, and 34.2% of cases in grade 1, 2, and 3, respectively. It isolated one microorganism in 23 patients and two microorganisms in nine patients. 

**Figure 3:**
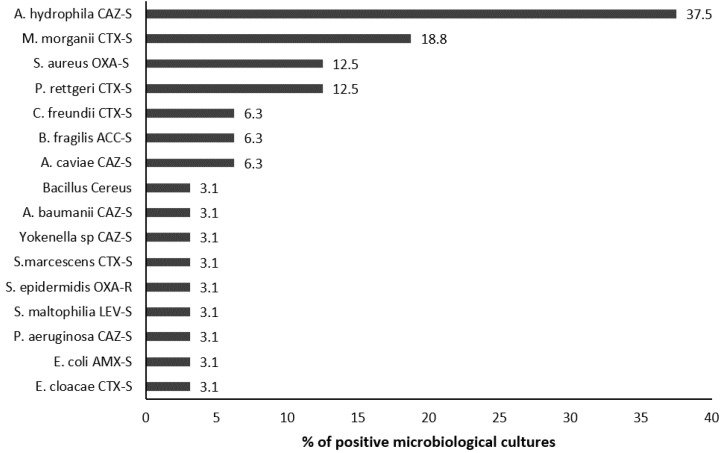
Isolated microorganisms from the local samples and blood cultures in patients with wound infection following snakebite. (The percentage is calculated based on the number of positive microbiological samples). CTX: cefotaxime, CAZ: ceftazidime, OXA: oxacillin, LEV: levofloxacin, AMX: amoxicillin, AAC: amoxicillin-clavulanate, S: susceptible.

**Figure 4:**
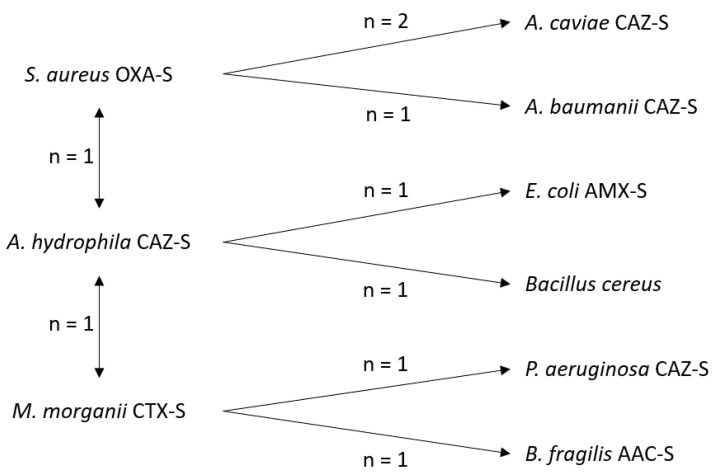
Coinfection patterns in patients with wound infection caused by microorganisms. CTX: cefotaxime, CAZ: ceftazidime, OXA: oxacillin, LEV: levofloxacin, AMX: amoxicillin, AAC: Amoxicillin-clavulanate, S: susceptible.

The isolated microorganisms were *A. hydrophila* susceptible to ceftazidime (CAZ-S) (37.5% of isolated microorganisms), *M. morganii* susceptible to cefotaxime (CTX-S) (18.8%), and *S. aureus* susceptible to oxacillin (OXA-S) and *P. rettgeri* CTX-S (12.5% each) (Figure 3). All microorganisms except one were wild type. However, they were resistant to amoxicillin–clavulanate in 82.9% of cases. The most isolated bacteria in patients with wound infection caused by two microorganisms were *S. aureus* OXA-S (4 cases), *A. hydrophila* CAZ-S (3 cases), and *M. morganii* CTX-S (3 cases) (Figure 4). Positive microbiological results were obtained from blood culture in two cases and local sampling in 30 cases. Antibiotic administration at admission was recorded in 11 cases with negative microbiological results (47.8%) and in 12 patients with positive microbiological results (37.5%) (*p* = 0.510).

### 2.5. Management of Wound Infection

The treatment of wound infection was based on local disinfection (all cases), abscess drainage (38/55 patients; 69.1%), and antibiotics (36/55 patients; 65.5%). The main antibiotics used were amoxicillin–clavulanate, metronidazole, cefotaxime, piperacillin-tazobactam, and ciprofloxacin in 32.7%, 16.4%, 16.4%, 13.9%, and 13.9% of cases, respectively. The length of antibiotic therapy was 7 days (IQR: 5–8 days). The treatment regimen was based on one antibiotic in 23/55 patients (41.8%), two in 12/55 patients (21.8%), and four in one patient. Nineteen patients (34.5%) did not receive antibiotics, and their management was only based on local measures. Among patients with positive microbiological cultures (*n* = 32), 23 received antibiotics (71.9%). The prescribed antibiotic was adequate for the isolated microorganism in eight cases (35%). Among patients with negative microbiological cultures (*n* = 23), 13 received antibiotics (56.5%). A third-generation cephalosporin or piperacillin-tazobactam was prescribed in 6/13 cases (46.2%).

Surgery was required in 48 patients (27.9%), and a necrosectomy was performed on 16 of them (33.3%). The delay from snakebite to surgery was 7 days (IQR: 5–9). Overall, the median hospital length of stay was 9 days (IQR: 6–13). It was 14 days (IQR: 11–23) in patients with wound infection and 7 days (IQR: 5–11) in those without (*p* < 0.001). Table 3 summarizes the management and outcome of our patients.

### 2.6. Independent Factors Associated with Wound Infection

The logistic regression model selected patient-related variables that were significantly associated with snakebite-related infection, namely, the grade of envenoming, necrosis, blisters, rhabdomyolysis, and thrombocytopenia. The independent factors associated with snakebite-associated infection were necrosis (*p* < 0.001, OR 13.15, 95% CI: 4.04–42.84), thrombocytopenia (*p* = 0.002, OR: 3.37, 95% CI: 1.59–7.16), and rhabdomyolysis (*p* = 0.046, OR: 2.29, 95% CI: 1.02–5.19) (Table 4).

## 3. Discussion

In the present study, the infection following SB was frequent (32% in our case series), and the patients at highest risk were those presenting with severe envenoming (grades 2 and 3). The main bacteria responsible for wound infection were *A. hydrophila* CAZ-S, *M. morganii* CTX-S, and *S. aureus* OXA-S and *P. rettgeri* CTX-S. 

In the literature, wound infection following snakebite occurs in 9–77% of patients [5,6,8,9,10,11]. The significant differences in the reported prevalence may be related to the case definitions used to characterize infection. A definite diagnosis can only be made when the responsible microorganism is isolated. In the remaining cases, infection is defined on a clinical basis, taking into account that clinical manifestations of local infection are quite similar to those induced by the venom toxins. Indeed, in clinical setting, it is difficult to differentiate local venom toxicity from wound infection, especially in severely envenomed patients. For this, we used the secondary appearance or worsening of local inflammatory symptoms as an indicator of the resolution or the stabilization of the toxic effect (related to envenoming) and the new appearance or worsening of local signs (related to infectious process). Overall, the secondary onset of signs evocative of wound infection suggest that the symptoms of snake envenoming are resolved and that local symptoms are related to a secondary event.

Documented wound infection combines local signs and positive microbiological cultures. However, in some cases, microbiological cultures remain sterile, and the diagnosis of infection is based solely on clinical evaluation. Moreover, initial empiric antibiotic therapy can result in negative microbiological cultures. Additionally, antibacterial activity in snake venoms have already been described in the literature [13,14,15,16]. Consequently, the prevalence of patients who developed wound infection secondary to snakebite could not be calculated except for those with positive microbiological cultures. Further studies should establish a more uniform set of criteria to define infection in snakebite envenoming and harmonize data that would allow for comparing studies.

In the present study, the main responsible bacteria were *A. hydrophila* CAZ-S, *M. morganii* CTX-S, and *S. aureus* OXA-S and *P. rettgeri* CTX-S. In the literature, the main involved bacteria in wound infection following snakebite are *A. hydrophila*, *M. morganii*, *S. marcescens*, Staphylococci, group D streptococci, *Clostridium*, *E. coli*, and *E. faecalis.* These bacteria have also been isolated from the mouth of viperid species [6,12]. Furthermore, *S. aureus* has rarely been isolated from the mouth of the snake, which suggests that these bacteria come from the patient’s skin instead of from the snake’s fangs. Therefore, strict disinfection of the bite site must be performed [17,18]. 

According to the Practice Guidelines for the Diagnosis and Management of Skin and Soft Tissue Infections [19], amoxicillin–clavulanate is recommended in bitten patients. In the present study, patients received antibiotics at admission in 36.6% of cases, mainly amoxicillin–clavulanate (92.1%). However, systematic antibiotic administration is questionable after a snakebite. Indeed, several studies showed that isolated Enterobacteriaceae following snakebite-related infection showed 60–70% resistance to amoxicillin–clavulanate. In our study, 82.9% of isolated bacteria were resistant to amoxicillin–clavulanate. Moreover, bacteria were susceptible to third-generation cephalosporins in 97% and to ciprofloxacin in 100% of cases [6,12,18,20,21,22]. So, systematic amoxicillin–clavulanate administration should not be advised, and we support the empiric use of third-generation cephalosporins in severe snake-bitten patients.

In our study, 82.9% of isolated bacteria were resistant to amoxicillin–clavulanate. Consequently, amoxicillin–clavulanate is not the best choice as empiric therapy in the case of snakebite. Surprisingly, physicians prescribed antibiotics at admission in 36.6% of cases, mainly amoxicillin–clavulanate (92.1%). This result shows the need to promote medical teams’ training for better and optimal snake-envenoming care in French Guiana. More surprisingly, the main antibiotic used in patients with wound infection was amoxicillin–clavulanate, and no antibiotic was prescribed in 34.5% of patients. In patients with positive microbiological cultures, the prescribed antibiotic was adequate for the isolated microorganism in only 35% of cases. In patients with negative microbiological cultures, antibiotics were also prescribed because of the complex nature of the oral microbiota in snakes, and bite wounds can harbor potential pathogens, many of which are anaerobes, which are difficult to document. In our study, the prescribed antibiotics in case of negative microbiological cultures were adequate for recommendations in only 46.2% of cases. This questions the usefulness of antibiotics in case of wound infections following snakebite and whether local measures are sufficient in some cases.

The results of this study argue in favor of the promotion of a protocol relating to the proper use of antibiotics in the event of a snakebite.

Snake oral cavities and fangs contain many pathogenic bacteria [12,21,22]. However, only some envenomed patients developed wound infection following the snakebite (32% in our study). Most of these patients were severely envenomed (grades 2 and 3) and presented rhabdomyolysis and thrombocytopenia. These signs reflect the prominent tissue damage probably related to the high amount of injected venom. Therefore, it is suggested that venom-induced skin and muscle damage is favorable for bacterial colonization and constitutes the bed of infection [23]. 

Our study has limitations. First, this was a mono-centric observational study. However, the Cayenne General Hospital provides care for more than two-thirds of the Guianese population [24]. Second, the involved bacteria were only identified in a limited number of cases (58.2%), having little clinical evidence of infection, probably because of a low inoculum, the involvement of anaerobic bacteria, antibiotics exposure, or the lack of microbiological sampling in some cases. The comparison of patients with and without isolated bacteria did not show a difference in prior antibiotic administration. Third, in cases without microbiological documentation, the diagnosis of infection was assessed clinically. This approach is approved by many authors [9,25,26,27], but further studies are needed to evaluate the diagnostic value of clinical and biological parameters to assess the diagnosis of wound infection following snakebite. Fourth, the snake identification was not very precise because the snake was not seen in many cases and can be known by different common names according to the geographic region [28,29]. However, it is well known that, in French Guiana and in the Amazon region, more than 90% of SB envenomings are caused by *B. atrox* [3,17]. Finally, we did not have the ability to measure time to infection resolution.

## 4. Conclusions

Wound infection following snakebite in French Guiana is frequent in grade 2 and 3 envenomed patients, especially those with necrosis, thrombocytopenia, and rhabdomyolysis. The main involved bacteria are *A. hydrophila* and *M. morganii*. Empirical antibiotics should be adapted to the most common isolated bacteria in this context and for at-risk patients. Our data support that the most appropriate empirical antibiotics are third-generation cephalosporins and that empirical amoxicillin–clavulanate should no longer be used in this context. Local disinfection, necrosectomy, and abscess drainage remain the essential measures for treating wound infection following snakebite.

## 5. Materials and Methods

The study is retrospective. It was conducted in the emergency department (ED) and the intensive care unit (ICU) of Cayenne General Hospital between 1 January 2016 and 31 July 2021. We included all patients hospitalized for snake envenoming regardless of the severity grade. The grade of envenoming was assessed in accordance with the recommendations of the international symposium on snakebites in French Guiana (Table 5) [17]. We excluded all patients with a history of snakebite without signs of envenoming.

Our hospital is a 510-bed general center that serves as a first-line medical center for an urban population of 100,000 inhabitants and as a referral center for a larger population coming from all the communities of French Guiana, accounting for almost 300,000 inhabitants [24]. 

### 5.1. Management of Snakebite Envenoming

The management of snake envenoming was based on the recommendations of the international symposium on snakebites in French Guiana [17]. All patients were checked for vaccination status and had a quick tetanus test. Anti-tetanus prophylaxis was administered accordingly. Some patients did not receive antivenom because there was no available antivenom in Cayenne, and snake-bitten patients were managed symptomatically until 2017 [2] or because of supply shortage. In 2017, the French Authority for Health recommended Antivipmyn Tri^®^ for the treatment of snake-envenomed patients in French Guiana. Moreover, in September 2017, an international symposium was held at Cayenne under the aegis of the French Regional Health Agency and the Pan American Health Organization [17]. The conclusions of this symposium illustrated the urgent need to ensure the accessibility of effective and safe polyvalent viperid antivenom in French Guiana. In the symposium, experts advised against systematic antibiotic administration in snake-envenomed patients.

### 5.2. Diagnosis and Management of Snakebite-Related Wound Infection

Wound infection following snakebite was defined by a secondary increase in local inflammatory symptoms or the appearance of pain, erythema, local warmth, swelling, lymphangitis, or purulence, independent of the grade of envenoming. In grade 2 or 3 envenomings, the wound infection was suspected in the case of local inflammatory symptoms independent of the timing of occurrence and was confirmed in the case of a positive microbiological culture.

The microbiological documentation of wound infection was based on positive microbiological cultures obtained from blood, local skin samples, and by culturing surgical specimens obtained during surgical debridement.

Local samples were subjected to Gram staining and cultured for bacterial growth. They were plated on nonselective blood agar and chocolate agar and cultured at 37 °C for 2–7 days, and the color and shape of the colonies were observed. Species identification was performed with API-20E and API-20NE systems (BioMérieux, Marcy L’Etoile, France). Blood cultures were performed using aerobic (Bact/ALERT FA plus) and anaerobic (Bact/ALERT FN plus) blood culture vials which were incubated in a BacT/ALERT 3D (BioMérieux, Marcy L’Etoile, France). All isolates were then identified using MALDI-TOF mass spectrometry (MaldiBiotyper 3.0, Bruker Daltonique, Marnes la Vallée, France). Antimicrobial susceptibilities of all isolates were determined by the disk diffusion method based on the definition of the Antibiogram Committee of the French Microbiology Society [30]. Susceptibility patterns were expressed as susceptible (S) or resistant (R) to cefotaxime (CTX), ceftazidime (CAZ), oxacillin (OXA), levofloxacin (LEV), or amoxicillin (AMX), according to the studied bacteria.

Upon admission to the emergency department or intensive care unit, a complete blood sample was taken, including, among other things, hemostasis, renal function, and creatinine kinase.

In all patients, we collected epidemiological and clinical data, including age and sex, the date and time of the bite, the anatomical site of the bite, the snake description, the grade of envenoming, and the clinical manifestations at admission and during the hospital stay. The complete cure of infection was obtained when clinical symptoms resolved.

### 5.3. Ethical Statement

Our study is retrospective and did not require individual consent according to the French law regarding research conforming to the norm MR-003 (JORF No. 0160, 13 July 2018, text No. 109). The protocol of antivenom administration and blood test dosages were approved by the hospital’s institutional review board (ref: UF3700/17′, version “b”—November 2016, revised on 5 March 2020). All patients were informed about the hospital protocol on the management of snakebite envenoming and were informed that the data collected would be used in research programs. Verbal consent was obtained from all patients or relatives (when patients were <18 years or unable to consent) and was reported in the medical file of the patient by the doctor in charge. In patients receiving antivenom, a completed form (blank form reference: Q11ADOC025 v01), including the patient data, the dosage, and the route of administration used, was completed and returned to the French National Agency for Drug Safety (ANSM: Agence Nationale de Sécurité de Médicaments). On this form, the physician in charge certified that the patient was informed about the drug use and that he would undertake to inform the ANSM of any adverse reaction. Our database was registered at the Commission National de l’Informatique et des Libertés (registration No. 2217025v0—27 February 2020), in compliance with French law on electronic data sources.

### 5.4. Definitions

Thrombocytopenia is defined by a platelet count <150 G/L. Defibrinogenation is defined by a fibrinogen level <1 g/L (normal value: 2–4 g/L). Rhabdomyolysis is defined by a CK level >500 UI/L (normal value: 39–308 UI/L). Coagulation disorders are defined by international normalized ratio >2 (normal value: 0.8–1.2), partial thromboplastin time >1.5, prothrombin time, and coagulation factors <60%. Renal failure is defined according to the KDIGO definition [31]. Adverse reactions to antivenom were reported to the French Agency for the Safety of Health Products and were classified as ‘mild’ (only cutaneous urticaria, pruritus) or ‘severe’ (bronchospasm, angioedema, hypotension, colic) [32].

### 5.5. Statistical Analysis

We created a data file with the patient’s and snake’s information, and we performed a descriptive analysis using Excel (2007) and IBM SPSS Statistics for Windows, version 24 (IBM Corp., Armonk, NY, USA). Results were reported as the number of patients for whom the data were recorded (Nb), the median and interquartile range (IQR), or numbers with percentages. Time is expressed as hours and minutes (hh:mm). To compare qualitative variables, we used the Fisher exact test. Continuous variables were tested for normality using Kolmogorov–Smirnov test (KS) and Shapiro–Wilk (SW) test. Variables with normal distribution were compared using independent sample *t*-test. Variables with non-normal distribution were compared using the Mann–Whitney U test.

Variables associated with snakebite-related infection at the 0.05 level by univariate analysis were entered into the stepwise logistic regression model. We calculated the odd ratio (OR) and the 95% confidence interval (95% CI). All statistical tests were two-tailed, and *p* ≤ 0.05 was considered significant.

## Figures and Tables

**Table 1:** Comparison of baseline characteristics in patients with and without wound infection following snakebite.

	Total Population		Wound Infection		Without Wound Infection		*p*
	Nb	Result	Nb	Result	Nb	Result	
Age (years)	172	41 (28–52)	55	46 (31.7–54.6)	117	40 (25.4–50.8)	0.340
Gender, male	172	119 (69.2%)	55	39 (70.9%)	117	80 (68.4%)	0.737
BMI	119	23.8 (21–26.9)	37	23.5 (20.3–26.9)	82	23.8 (21.3–26.9)	0.462
**Medical history**	**172**	**42 (24.4%)**	**55**	**12 (21.8%)**	**117**	**30 (25.6%)**	
Arterial hypertension	172	13 (8%)	55	2 (3.6%)	117	11 (9.4%)	0.182
Alcohol abuse	172	6 (3%)	55	1 (1.8%)	117	5 (4.3%)	0.413
**Snake bite (SB)**							
Time from SB to hospital (hh:mm)	171	9:00 (2:02–21:00)	55	12:00 (3:30–22:58)	116	7:07 (1:43–19:03)	0.064
Identification of the snake	172	78 (45.3%)	55	21 (38.2%)	117	57 (48.7%)	0.195
**Anatomic site of the bite**							
Upper limb	172	34 (19.8%)	55	10 (18.2%)	117	24 (20.5%)	0.328
Lower limb	172	137 (79.7%)	55	44 (80%)	117	93 (79.5%)	
Head	172	1 (0.6%)	55	-	117	1 (0.8%)	-
**Grade of envenoming**							
Grade 1	172	74 (43%)	55	14 (25.5%)	117	60 (51.3%)	Reference
Grade 2	172	60 (34.9%)	55	21 (38.2%)	117	39 (33.3%)	0.035
Grade 3	172	38 (22.1%)	55	20 (36.4%)	117	18 (15.4%)	<0.001
**Clinical presentation at admission**							
Edema	172	165 (95.9%)	55	55 (100%)	117	110 (94%)	0.064
Local hemorrhage	172	22 (12.8%)	55	8 (14.5%)	117	14 (12%)	0.637
Necrosis	172	21 (12.2%)	55	17 (30.9%)	117	4 (3.4%)	<0.001
Blisters	172	22 (12.8%)	55	14 (25.5%)	117	8 (6.8%)	0.001
Pain	171	165 (96.5%)	54	53 (98.1%)	117	112 (95.7%)	0.424
Temperature (°C)	165	36.9 (36.5–37.3)	49	37 (36.6–37.4)	116	36.9 (36.5–37.3)	0.045
Shock	172	2 (1.2%)	55	1 (1.8%)	117	1 (0.9%)	0.583
Acute renal injury	172	28 (16.3%)	55	12 (21.8%)	117	16 (13.7%)	0.177
Time to normal renal function (days)	28	5 (28–10)	12	4 (2–23)	16	6 (2–10)	0.958
Systemic hemorrhage (SH)	172	24 (14%)	55	7 (12.7%)	117	17 (14.5%)	0.750
Time from SB to SH (hours)	24	5 (0–24)	7	4 (1–8)	17	7 (0–24)	0.125
**Biological parameters at admission**							
Fibrinolysis	172	151 (87.8%)	55	45 (81.8%)	117	106 (90.6%)	0.101
Thrombocytopenia	172	49 (28.5%)	55	25 (45.5%)	117	24 (20.5%)	0.001
Hemolysis	172	38 (22.1%)	55	15 (27.3%)	117	23 (19.7%)	0.262
Rhabdomyolysis	172	54 (31.4%)	55	26 (47.3%)	117	28 (23.9%)	0.002
White blood count (/mm^3^)	167	10.4 (8.3–13.6)	53	12 (9.4–14.6)	114	10.2 (8–13.2)	0.016
C-reactive protein (mg/L)	153	8.7 (2.8–32.2)	46	27.6 (5.6–83.2)	107	6.4 (2.7–19.6)	0.002
Procalcitonin (µmol/L)	10	0.1 (0–0.5)	0	-	10	0.1 (0–0.5)	-

Nb: number of patients for whom the data were recorded, SB: snakebite, BMI: body mass index, SH: systemic hemorrhage.

**Table 2:** Clinical and therapeutic parameters recorded in patients with wound infection.

	Nb	Result
Abscess	55	38 (69.1%)
Necrotizing fasciitis	55	9 (16.4%)
Cellulitis	55	12 (21.8%)
Isolated microorganism	55	32 (58.2%)
Time from SB to WI (days)	55	6 (3–8)
Antibiotic duration for WI (days)	36	7 (5–8)

Nb: Number of patients for whom the data were recorded, WI: wound infection.

**Table 3:** Comparison of the management strategy and outcome of patients with and without wound infection following snakebite.

	Total Population		Wound Infection		Without Wound Infection		*p*
	Nb	Result	Nb	Result	Nb	Result	
Renal replacement therapy	172	9 (5.2%)	55	4 (7.3%)	117	5 (4.3%)	0.410
Noradrenaline	172	2 (1.2%)	55	1 (1.8%)	117	1 (0.9%)	0.583
Mechanical ventilation	172	3 (1.7%)	55	2 (3.6%)	117	1 (0.9%)	0.194
Antibiotics at admission	172	63 (36.6%)	55	22 (40%)	117	41 (35%)	0.391
Antivenom therapy (AV)	172	115 (66.9%)	55	32 (58.2%)	117	83 (70.9%)	0.097
Time from SB to AV	83	9:00 (5:22–20:40)	22	10:00 (6:00–19:45)	61	9:00 (5:15–21:00)	1
Adverse reaction to AV	116	20 (17.2%)	33	7 (21.2%)	83	13 (15.7%)	0.284
Transfusion	172	17 (9.9%)	55	9 (16.4%)	117	8 (6.8%)	0.51
Surgery	172	48 (27.9%)	55	43 (78.2%)	117	5 (4.3%)	<0.001
Necrosectomy	48	23 (47.9%)	43	21 (48.8%)	5	2 (40%)	0.476
Time from SB to surgery (days)	48	7 (5–9)	43	7 (5–9)	5	5 (3–8)	0.865
Length of stay in ICU (days)	138	3 (3–5)	43	4 (3–8)	95	3 (3–4)	0.370
Length of hospital stay (days)	172	9 (6–13)	55	14 (11–23)	117	7 (5–11)	<0.001

Nb: Number of patients for whom the data were recorded, SB: snakebite, AV: antivenom.

**Table 4:** Factors associated with wound infection after snake envenoming (multivariate analysis).

			Univariate Analysis	Multivariate Analysis		
Variable	Wound Infection (*n* = 55)	Without Wound Infection (*n* = 117)	*p*	OR	95% CI	*p*
Blisters	14 (25.5%)	8 (6.8%)	0.001	2.812	0.909–8.697	0.073
Thrombocytopenia	25 (45.5%)	24 (20.5%)	0.001	3.374	1.594–7.163	0.002
Necrosis	17 (30.9%)	4 (3.4%)	<0.001	13.15	4.042–42.841	<0.001
Rhabdomyolysis	26 (47.3%)	28 (23.9%)	0.002	2.294	1.015–5.187	0.046
Grade 2	21 (38.2%)	39 (33.3%)	0.035	1.947	0.822–4.615	0.130
Grade 3	20 (36.4%)	18 (15.4%)	<0.001	0.875	0.263–2.916	0.828

OR: odd ratio, 95% CI: 95% confidence interval.

**Table 5:** Severity grading scale of snake envenoming [17].

		Grade		
		1 (Mild)	2 (Moderate)	3 (Severe)
Coagulation disorder		present	present	present
Local symptoms	Pain	present	present	present
	Swelling	Not exceeding elbow or knee	Exceeding elbow or knee	Beyond the root of the limb
	Blister	absent	present	present
	Necrosis	absent	absent	present
Local or systemic bleeding		absent	present	present
Systemic manifestations (Hypotension, renal injury, coma, respiratory failure, …)		absent	absent	present

## Data Availability

Data supporting reported results can be found with the corresponding author.
